# Neural Responses of Benefiting From the Prosocial Exchange: The Effect of Helping Behavior

**DOI:** 10.3389/fpsyg.2021.606858

**Published:** 2021-03-04

**Authors:** Daniele Olivo, Andrea Di Ciano, Jessica Mauro, Lucia Giudetti, Alan Pampallona, Katharina M. Kubera, Dusan Hirjak, Robert Christian Wolf, Fabio Sambataro

**Affiliations:** ^1^Department of Neuroscience (DNS), University of Padua, Padua, Italy; ^2^Department of Medicine (DAME), University of Udine, Udine, Italy; ^3^Fondazione Giancarlo Quarta, Milan, Italy; ^4^Center for Psychosocial Medicine, Department of General Psychiatry, Heidelberg University, Heidelberg, Germany; ^5^Department of Psychiatry and Psychotherapy, Central Institute of Mental Health, Medical Faculty Mannheim, Heidelberg University, Mannheim, Germany; ^6^Padua Neuroscience Center, University of Padua, Padua, Italy

**Keywords:** prosocial exchange, practical help, effort appreciation, fMRI, helping behavior, theory of mind

## Abstract

Prosocial behavior is critical for the natural development of an individual as well as for promoting social relationships. Although this complex behavior results from gratuitous acts occurring between an agent and a recipient and a wealth of literature on prosocial behavior has investigated these actions, little is known about the effects on the recipient and the neurobiology underlying them. In this study, we used functional magnetic resonance imaging to identify neural correlates of receiving prosocial behavior in the context of real-world experiences, with different types of action provided by the agent, including practical help and effort appreciation. Practical help was associated with increased activation in a network of regions spanning across bilateral superior temporal sulcus, temporoparietal junction, temporal pole, and medial prefrontal cortex. Effort appreciation was associated with activation and increased task-modulated connectivity of the occipital cortex. Prosocial-dependent brain responses were associated with positive affect. Our results support the role of the theory of mind network and the visual cortices in mediating the positive effects of receiving gratuitous help. Moreover, they indicate that specific types of prosocial behavior are mediated by distinct brain networks, which further demonstrates the uniqueness of the psychological processes underlying prosocial actions.

## Introduction

Prosocial behaviors are a set of acts aimed directly at benefiting people other than oneself, and they entail helping, comforting, cooperation, and kindness (Batson, 2012). This definition comprises a broad number of behaviors ranging from providing support in response to an immediate physical threat to someone else, to helping someone to carry the grocery, or to listening supportively to the other person’s predicaments. Although by definition, prosocial behaviors are altruistic in nature and sometimes they may have negative consequences for the person that delivers them, they have positive physical and mental long-term effects (Inagaki and Eisenberger, 2016; Wang et al., 2020). Indeed, these behaviors, including volunteering, have been associated with lower levels of depression (Kim and Pai, 2010) and greater mental well-being and satisfaction in life (Tabassum et al., 2016). Not only society but also the individual can benefit from carrying out this behavior. It is noteworthy that kind behavior predicted an increase in subjective happiness, which can mediate positive effects on well-being as well as on social relationships, and this can play an adaptive role (Otake and Fredrickson, 2012).

Although the agent, who performs the actions, and the recipient, who benefits from them, have both fundamental roles in prosocial behavior, the literature on the neural effects of this interaction on the recipient is quite limited. Indeed, receiving help can cause mixed moral emotions (Fisher et al., 1982), including gratitude, indebtedness, and guilt, and the type of elicited psychological responses can affect social exchanges and ultimately interpersonal relationships (Algoe, 2012). In particular, gratitude is a positive interpersonal emotion deriving from the combination of the appreciation of the action and the pleasure of enjoying the action itself (McCullough, 2002). The recipient most frequently experiences this emotion when the agent is thought to have benevolent intentions (Tsang, 2006), to give gratuitously (Watkins et al., 2006), and to sustain a cost and that the action that is received is valuable (Tsang, 2006). Indebtedness is more often a negative emotion associated with discomfort, which stems from the perceived need to reciprocate another to restore equity in social interactions. Guilt may also arise from a prosocial interaction. This is a negative emotion associated with violating moral codes, which is associated with indebtedness and reflects the expectation of failing to reciprocate for timing (e.g., too early or too late) or inability to do it properly (Layous et al., 2017). Although traditionally studied as separate entities, these emotions tend to co-exist in social exchanges. Several factors can contribute to the experience of these emotions with respect to a prosocial interaction: traits of personality, such as self-focused attention, can favor indebtedness and particularly in those individuals more prone to perceive themselves as social objects and to have greater negative self-evaluation (Mathews and Green, 2010); cultural determinants play also a role, with individuals from Eastern Asia feeling greater indebtedness relative to Western people (Hitokoto, 2016).

Furthermore, within prosocial behaviors, the type of gratuitous help, although similar for intent and purpose, may differ in the level of autonomy of the receiver (Pierce et al., 1997). For example, in a medical relationship, a patient can let the doctor decide about the treatment or make an informed decision, where the doctor has only a supportive function in the decision. These two polar approaches are *practical help and effort appreciation.* Practical help is delivered when the agent performs a behavior directly on the source of the problem, and the receiver passively benefits from material aid, dependency-oriented help, e.g., helping in carrying heavy bags or providing a meal. On the other hand, *effort appreciation* is the conduct whereby the agent values the efforts of the receiver at resolving a specific issue, autonomy-oriented help, e.g., commenting positively on quitting smoking or having a healthier diet. Although both types of aid can be perceived as prosocial and positive, the role of the receiver is different. In the practical help condition, the receiver passively benefits from an immediate gratuitous help/aid, and this is generally associated with gratitude and/or indebtedness (Lane and Anderson, 1976). In the effort appreciation, the receiver actively performs an action/behavior, and a positive feedback strategy is delivered; again, this is associated with positive affect and its effects can foster subsequent learning that is crucial during the development stages of life (Qu and Zelazo, 2007) as well as for promoting changes linked with healthier lifestyle (Young, 2014).

Recent studies have started to identify the brain networks underlying prosocial behaviors (Telzer et al., 2011; Lockwood et al., 2016; Will et al., 2016; Lamm et al., 2019). The medial and lateral parts of the prefrontal cortex and the anterior cingulate have been associated with the computation of the cost-benefit value of an action (Apps and Ramnani, 2014) and its social and moral assessment as well as with mediating the cognitive conflict between personal interests and the other’s needs (Cohen and Lieberman, 2010). The medial prefrontal cortex (mPFC), temporoparietal junction (TPJ), and posterior superior temporal sulcus (pSTS) have been implicated in mentalizing and perspective taking (Mitchell et al., 2005; Decety and Lamm, 2007; Behrens et al., 2008). In particular, right TPJ has been implicated in the inference of other’s effort during movement that is important in estimating the cost of helping, and together with its role in reorienting attention can contribute to higher-level social cognition (Mizuguchi et al., 2016). Additionally, the default mode network (DMN), which is supposed to mediate self-referential processes such as autobiographical navigation, daydreaming, or emotional introspection (Raichle et al., 2001), has been implicated in the other-referential thinking and social behavior (Andrews-Hanna, 2012; Mars et al., 2012). Indeed, a very recent study on indebtedness found the engagement of a network encompassing the Theory of Mind (i.e., DMPFC and TPJ) as well as the DMN during an interactive game with different conditions of reciprocity (Gao et al., 2020). Furthermore, different regions of sensory motor and DMN may be recruited in processing comments on the self. Positive social feedback in children during a social evaluation paradigm was associated with increased activation in occipital areas, post-central, striatum, and cingulate cortex (Achterberg et al., 2016). Subjects receiving positive evaluative feedback on the self during a verbal social approval task investigating personal actions in socio-moral context showed increased activation in pre- and post-central regions, occipito-temporal (mainly, lingual gyrus), and cingulate cortex (van Schie et al., 2018).

In the present study, we used functional magnetic resonance imaging (fMRI) to characterize the neural correlates of receiving prosocial behavior. To have greater ecological validity, to elicit the brain responses associated with this prosocial behavior, we used vignettes portraying practical help and effort appreciation that were depicted in real life situations (Knutson et al., 2010). To avoid the type of help by valence interaction, we limited our study to situations that elicited positive affect. Our first goal was to characterize the neural correlates underlying receiving gratuitous support. Secondly, we wanted to identify the differential mechanisms underlying specific types of aid. Third, given the association of positive affect and altruism, we tested whether brain responses underlying received prosocial behavior were associated with positive affect. Based on previous literature, we anticipate that practical help will elicit greater activation of the ToM and DMN networks (Mizuguchi et al., 2016; Gao et al., 2020) for their role in mediating the direct costs of social interaction and indebtedness. On the other hand, effort appreciation, which entails social approval for the self, is expected to be associated with a prefrontal-cingulate network along with brain regions involved in visual perception as suggested by previous studies on social reward (Achterberg et al., 2016; Makowski et al., 2016). In addition, we expect that the brain activations associated with helping conditions will be associated with a greater propensity to positive affect during social interactions.

## Materials and Methods

### Subjects

Thirty right-handed Italian native speakers (11 males; age range between 19 and 33 years) with reported normal or corrected-to-normal vision participated in the study. Exclusion criteria included (1) any history of substance abuse or dependence (excluding nicotine); (2) head trauma with loss of consciousness; (3) any current medical or neurological disease; (4) any personality disorder; (5) documented intellectual impairment; (6) any contraindications for MRI scan; and (7) excessive head motion during MRI scan.

All research participants gave written informed consent to the study, which was approved by the Institutional Review Board of the University of Parma (UNIPRMR750v1) and conducted in accordance with the principles expressed in the Declaration of Helsinki.

### Procedure

During the functional magnetic resonance imaging (fMRI) acquisition, each participant performed a social interaction task.

#### Stimuli

**FIGURE 1:**
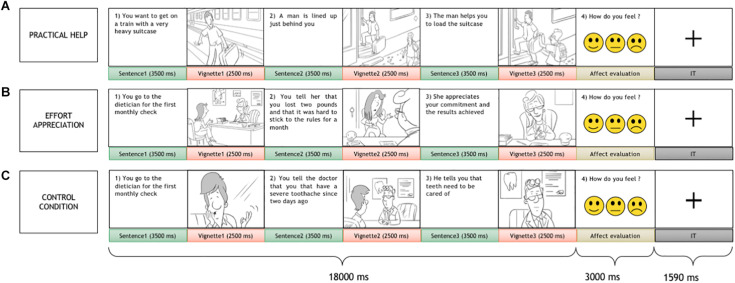
The social interaction task. Each trial included two parts: the display of a social interaction scene composed of three sentences alternated with three vignettes and an affect rating task. A variable duration intertrial interval was presented in between trials. The participant was asked to identify himself/herself with the gray-haired person, the receiver, who interacted with another person, the agent. Three social interaction conditions were presented: two helping conditions, practical help **(A)**, effort appreciation **(B)**, and a non-helping control condition **(C).**

Each trial consisted of two parts: a passive presentation of social interactions and an active affect rating task. At the beginning of each trial, a series of 3 vignettes and 3 sentences in between, depicting a social interaction between two people were presented. In the vignettes, a gray-haired character, the receiver, who the participant was requested to identify with, and another person, the agent, interacted in everyday life scenes. The sentences were short descriptive passages (max 2 lines) in between vignettes with similar length and complexity across conditions, meant to clarify and link the vignettes to help in their mental representation. Each trial portrayed a social interaction, including helping (practical help and effort appreciation) and non-helping (control) conditions, where the receiver performed an action and interacted with other people. In practical help, the receiver, who faces a task, is helped unconditionally by the agent; on the other hand, in effort appreciation, the receiver willingly performs an action/behavior to pursue his/her goals and in doing so is valued by the agent. The non-helping control condition, similar in visual, and conceptual complexity to the social interaction conditions, included a situation where the receiver accomplished a result without direct communication or behavioral intervention by the agent (for more details see Figure 1). After the social interaction, three smileys in a row displaying happy, neutral, and sad faces were presented, and the subject was asked to rate his/her affect relative to the social interaction by a button press. Visual complexity for vignettes, action content, and word length for sentences did not differ across helping conditions (see Supplementary Materials for details).

#### Design

Ninety pseudorandomized trials were presented; 60 non-repeating helping scenarios depicting effort recognition and practical help conditions (30 each) matched for visual and textual complexity, alternated with 30 non-helping control trials that repeated the scenarios of the helping conditions. Each condition trial consisted of a sequence of three 3500-ms sentence slides, alternated with three 2500-ms vignettes, for a total duration of 18000 ms, and was followed by an affect rating task lasting for 3000 ms (Figure 1). In between the trials, a crosshair was presented for 1590 ms. Before the scan, participants were trained with a brief mock session, during which they were explicitly instructed to empathize with the receiver in the scene.

### Image Acquisition

All MRI images were acquired on a 3.0 T scanner (GE Discovery MR750, Local Health Unit of Parma). For each subject, structural T1-weighted images were acquired by 3D-MPRAGE sequence with the following parameters: TR = 9700 ms, TE = 3.97 ms, FOV = 256 × 256 mm2, voxel size = 0.5 × 0.5 × 0.9 mm3. fMRI scan was performed using a gradient-echo-planar imaging (GE-EPI) sequence with the following parameters: TR = 2000 ms, TE = 30 ms, flip angle = 90°, FOV = 240 × 240 mm^2^, voxel size = 3.2 × 3.2 × 3.5 mm^3^. The social interaction task scan comprised 338-volumes per each run.

### Imaging Preprocessing

The preprocessing pipeline was run in DPABI^1^. The scans were visually inspected by FS and DO to exclude artifacts or lesions. All functional and anatomical images were reoriented to set the origin to the anterior commissure and the horizontal axis parallel to the AC-PC line. Then, the functional images were realigned (a least-squares approach and a six-parameter spatial transformation) to correct for head motion and co-registered to the individual structural scan. The anatomical images were segmented and normalized into the Montreal Neurological Institute standard space using the MNI152 template and the estimated parameters were applied to all functional images that were resampled to a 3 × 3 × 3 mm^3^ voxel size. Finally, images were smoothed using a 6 mm full width at half maximum Gaussian kernel to increase the signal-to-noise ratio and to compensate for residual anatomical variation across subjects.

### Imaging Analysis

The data were analyzed using a general linear model (GLM) with statistical parametrical mapping (SPM12^2^). The task was modeled with four block regressors: three regressors for the social interaction condition (practical help, effort appreciation, and control) and one for the affect rating task. Each block was modeled with a box-car and convolved with a canonical hemodynamic response function. Six-motion parameters estimated during realignment were included as nuisance covariates. In the first level analyses, whole-brain t-contrast maps of each interacting condition relative to a non-helping control (i.e., practical help greater than control condition and effort appreciation greater than control condition but not the reverse contrast were tested) as well as a direct comparison between social interaction conditions (effort appreciation greater than practical help; practical help greater than effort appreciation) were computed for each subject. In the second-level analyses, individual contrasts were entered in random-effect group analysis (Friston et al., 1999) and one-sample *t*-test spatial maps were estimated for each contrast.

Psychophysiological interaction (PPI) analyses were performed to identify task-dependent changes in connectivity between the brain regions identified in the activation analyses. We used the results from group analyses as functional localizer to draw the seeds for the PPI: 5-mm-radius spherical masks centered on the peak coordinates of rTPJ (peak xyz = 63, –9, 12) (Shamay-Tsoory, 2011; Morelli et al., 2014) for practical help and lingual gyrus (peak xyz = 3, –84, –9) (Fitzgerald et al., 2013; Schiffer et al., 2014) for effort appreciation, respectively, were created. The first eigenvariate of subject time-courses was extracted from these seeds, mean-centered, high-pass filtered, and deconvolved. A general linear model with three regressors was computed for each subject for each PPI using: a physiological regressor (the time course from the seed), a psychological regressor (multiple conditions designed in the social interaction task), and a psychophysiological interaction term, calculated as the cross-product of the physiological and psychological regressors. Individual PPI contrasts were entered into random-effects one-sample *t*-tests testing for increased and reduced brain connectivity in the helping relative to the control condition.

A whole-brain family-wise error-corrected threshold of *p* < 0.05 at the cluster level with a cluster-defining threshold of uncorrected *p* < 0.001 at the voxel-level was applied using Gaussian random field theory in SPM. The minimum cluster size for the activation analyses was 118 voxels in practical help > control, 926 voxels in effort recognition > control, 97 voxels in practical help > control, and 206 voxels in effort recognition > practical help contrasts. For the PPIs, 176 voxels for negative interaction for practical help, 206 and 260 voxels for positive and negative interaction for effort recognition, respectively.

To identify shared functional connectivity networks that are modulated by prosocial conditions, we created conjunction maps of the statistical maps of PPI effects for practical help and effort appreciation relative to controls condition. Statistical maps were thresholded with *p* < 0.001 so that the resulting conjoint probability of the conjunction maps was *p* < 10-6 (Sambataro et al., 2012).

To infer the mental processes associated with activation and connectivity in specific brain regions from published fMRI studies, a functional decoding approach implemented in Neurosynth database^3^ was used. Briefly, the contrast maps for each prosocial condition and PPI was uploaded to the database and voxel-wise Pearson’s correlations with each of 400 topic-based meta-analysis maps were carried out (see Yarkoni et al., 2011 for details on Neurosynth methods) (Yarkoni et al., 2011). The 10 highest correlated items for each map are reported in the Supplementary Materials (Supplementary Tables S1 and S2).

### Behavioral Analysis

The behavioral responses, recorded during the affect evaluation part of the fMRI task for each social interaction condition, were averaged for each participant and analyzed offline. The altruistic nature of the help received in the scenes was expected to elicit positive affective responses. For this reason, affect responses were binarized into positive and negative responses, this latter group including button presses indicating negative and neutral affect. Positive responses showed high negative skewness for helping conditions and positive skewness for non-helping conditions (Supplementary Figure S2A), conversely, negative responses showed high positive skewness for helping conditions and negative skewness for non-helping conditions (Supplementary Figure S2B). The proportion of positive responses across social interaction conditions was compared using chi-square tests. To assess the tendency to respond positively to helping behavior, we used a signal-detection theory approach (Green and Swets, 1966). We indexed performance according to participants’ ability to identify the altruistic nature of a scene based on a positive affect response relative to a non-altruistic condition. Positive responses to helping conditions were considered hits, negative/neutral responses to the non-altruistic condition were considered correct rejects, and positive responses to the non-altruistic condition were considered false alarms. We calculated C-score by averaging the *z*-score that corresponds to the hit rate and the *z*-score that corresponds to the false alarm rate (Green and Swets, 1966). This index reflects the subject’s response strategy, which is in our case the inner propensity to provide a positive affect response to social interactions. One outlier with performance more than three standard deviations away from the mean positive affect ratings for both prosocial conditions was excluded from these analyses. To test whether the participant’s sex could influence the affect ratings we used three-way ANOVAs with participant’s sex, task condition, recipient’s sex as predictors, and positive affect ratings as the dependent variable.

To control for personality traits, participants completed also the Big Five Questionnaire (BFQ) for the measurement of the Big Five-Factor Model (which includes the factors Extraversion, Agreeableness or Friendliness, Conscientiousness, Emotional Stability or Neuroticism, and Intellect or Openness to Experience) (Caprara et al., 1993).

### Brain Behavior Correlations

The first eigenvariate of the contrast images for each social interaction condition for each subject within an 8-mm-radius sphere centered on the peak clusters/subclusters was extracted from significant clusters at the group level. For each social interaction condition, brain responses were correlated with the respective C-score, indicating the propensity to provide a positive response as well as with each BFQ dimension. Bonferroni correction was used to correct for multiple comparisons.

## Results

### Behavioral Results

The affect following practical help (80.5 ± 14.0%, mean ± SD) as well as effort appreciation (82.4 ± 16.3%) trials were rated more positively than in control condition (15.3 ± 9.5%; see Supplementary Materials for the distribution of response type per condition across subjects) and this difference was significant for both prosocial interaction conditions (practical help > control condition, χ^2^ = 87.1, *p* < 0.001; effort appreciation > control condition, χ^2^ = 89.8, *p* < 0.001), but not between them (*p* > 0.1). The C-scores for prosocial conditions indicated a greater propensity to attribute to both of them (practical help, 0.08 ± 0.33; effort appreciation, 0.03 ± 0.41) a positive valence relative to the control condition. No difference in propensity was present between conditions (*p* > 0.1). We did not find any effect of participant’s-responder’s sex match/mismatch (*p* > 0.1; see Supplementary Material S.6. for further details).

### Social Interaction Task

#### Main Effect of Social Interaction Condition

**FIGURE 2:**
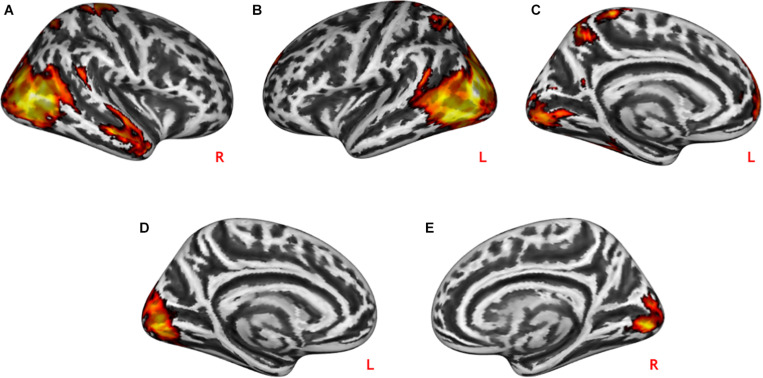
Neural effects of social interactions. Practical help was associated with increased activation in the medial prefrontal as well as temporo-parieto-occipital regions in both the right **(A)** and left **(B,C)** hemispheres. Effort appreciation was associated with increased left **(D)** and right **(E)** occipital activation. Statistical probability maps are rendered on an MNI template with a threshold of voxel-wise *p* < 0.001 and FWE-corrected *p* < 0.05 at the cluster level. MNI, Montreal Neurological Institute; FWE, family-wise error. L and R indicate left and right brain hemisphere, respectively.

During practical help, the posterior region of the middle temporal-occipital cortex (rMTOC, *z* = 5.78, *p* = 3.7^∗^10^–9^), posterior middle temporal gyrus (pMTG), left middle occipital cortex (lMOC, *z* = 6.18, *p* = 3.3^∗^10^–10^), right temporal gyrus (rTG, *z* = 4.71, *p* = 1.3^∗^10^–6^), precuneus (*z* = 4.65, *p* = 1.7^∗^10^–6^), medial postcentral gyrus (*z* = 4.62, *p* = 1.9^∗^10^–6^) and medial prefrontal cortex (mPFC, *z* = 4.06, *p* = 2.4^∗^10^–5^) showed greater activation relative to control condition (see Table 1 and Figures 2A–C). Effort appreciation revealed greater activation of the visual cortex relative to the control condition, particularly the lingual gyrus (*z* = 5.62, *p* = 9.8^∗^10^–9^) (see Table 1 and Figure 2D,E).

**TABLE 1:** Montreal Neurological Institute coordinates of weighted centers and peaks of clusters from the two main contrasts: practical help > control condition and effort appreciation > control condition.

**Cluster location**	***z*-score**	**Peak coordinates**			***K*-size**
		**X**	**Y**	**Z**	
***Practical help > control condition***					
Middle occipital cortex	6.18	–45	–75	15	1592
Middle temporal-occipital cortex	5.78	54	–66	9	995
Middle temporal-occipital cortex	5.41	45	–60	12	995
Middle temporal gyrus	4.71	63	–9	12	118
Precuneus	4.65	3	–57	45	314
Postcentral gyrus medial segment	4.62	–9	–42	63	182
Medial prefrontal cortex	4.06	–6	54	–9	193
***Effort appreciation > control condition***					
Lingual gyrus	5.62	3	–84	–9	926

#### Between Conditions Comparisons

**FIGURE 3:**
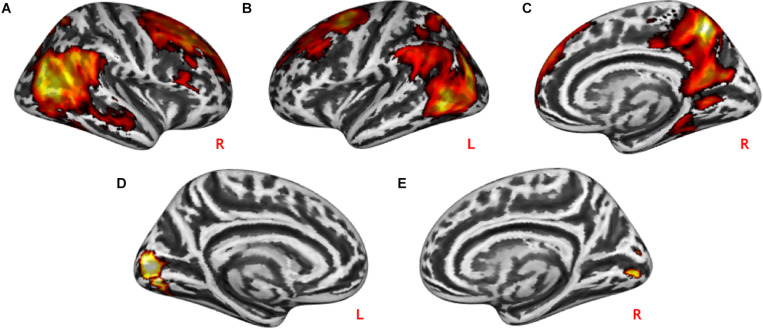
Neural differences between social interaction conditions. Practical help was associated with greater activation in the prefrontal cortex, temporo-parieto-occipital region including TPJ in both the right **(A,C)** and left **(B)** hemispheres relative to effort appreciation. Effort appreciation was associated with increased left **(D)** and right **(E)** occipital activation relative to practical help. Statistical probability maps are rendered on an MNI template with a threshold of voxel-wise *p* < 0.001 and FWE-corrected *p* < 0.05 at the cluster level. MNI, Montreal Neurological Institute; FWE, family-wise error; TPJ, temporoparietal junction. L and R indicate left and right brain hemisphere, respectively.

Practical help showed greater activation in posterior regions of the temporo-occipital cortex (lMTOC, *z* = 7.03, *p* = 1.0^∗^10^–12^, rMOTC *z* = 6.94, *p* = 2.0^∗^10^–12^) comprising temporoparietal junction (TPJ) and inferior parietal lobule (IPL) but also middle frontal gyrus (*z* = 6.04, *p* = 7.7^∗^10^–10^), superior frontal gyrus (*z* = 5.90, *p* = 1.9^∗^10^–9^) and precuneus (*z* = 7.51, *p* = 2.9^∗^10^–14^) relative to effort appreciation (see Table 2 and Figures 3A–C). The visual cortex, particularly calcarine (*z* = 4.48, *p* = 3.8^∗^10^–6^) and lingual gyrus (*z* = 4.24, *p* = 1.1^∗^10^–5^), showed greater activation during effort appreciation compared to practical help (see Table 2 and Figures 3D,E).

**TABLE 2:** Montreal Neurological Institute coordinates of weighted centers and peaks of clusters between the two main contrasts: practical help > effort appreciation and effort appreciation > practical help.

**Cluster location**	***z*-score**	**Peak coordinates**			***K*-size**
		**X**	**Y**	**Z**	
***Practical help > effort appreciation***					
Precuneus	7.51	9	–57	48	1744
Middle temporal gyrus	7.03	–54	–63	0	1236
Middle occipital gyrus	6.94	45	–66	15	1528
Fusiform gyrus	6.27	–30	–39	–15	118
Middle frontal gyrus	6.04	–24	3	54	713
Superior frontal gyrus	5.90	24	48	30	1766
Inferior temporal gyrus	5.10	48	–48	–21	97
Posterior cingulate gyrus	4.60	–15	–57	6	151
***Effort appreciation > practical help***					
Calcarine gyrus	4.48	–9	–81	3	206
Lingual gyrus	4.24	–12	–75	–6	206

#### Psychophysiological Interactions

**FIGURE 4:**
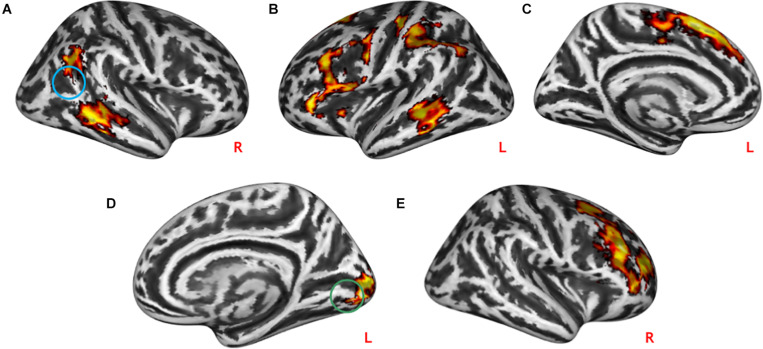
Task-modulated connectivity during social interactions. Practical help was associated with reduced task-modulated connectivity of the right TPJ with the prefrontal cortex, and temporoparietal regions in both the right **(A)** and left **(B,C)** hemispheres. During effort appreciation, the task-modulated connectivity of the lingual gyrus increased with the occipital cortex **(D)** and decreased with the right prefrontal cortex **(E)**. Statistical probability maps are rendered on an MNI template with a threshold of voxel-wise *p* < 0.001 and FWE-corrected p < 0.05 at the cluster level. The approximate location of the seeds is represented by colored circles (TPJ, blue; lingual gyrus, green). MNI, Montreal Neurological Institute; FWE, family-wise error; TPJ, temporoparietal junction. L and R indicate left and right brain hemisphere, respectively.

The task-modulated connectivity between rTPJ with the left medial and inferior frontal gyrus (lMFG *z* = –4,56, *p* = 2.6^∗^10^–6^, lIFG *z* = –4.51, *p* = 3.2^∗^10^–6^), left post-central cortex (lPG *z* = –4.13, *p* = 1.8^∗^10^–5^), and bilateral MTG (lMTG *z* = –4.47, *p* = 3.9^∗^10^–6^ and rMTG *z* = –4.39, *p* = 5.7^∗^10^–6^) was decreased during practical help relative to control condition (see Table 3 and Figures 4A–C). During effort appreciation, the task-modulated connectivity of the medial part of the lingual gyrus (*z* = 4.42, *p* = 5.0^∗^10^–6^) increased with the lateral part of this region along and decreased with the right (rMFG *z* = –4.69, *p* = 1.4^∗^10^–6^) and anterior cingulate cortex (ACC *z* = –4.04, *p* = 2.7^∗^10^–6^) relative to control condition (see Table 3 and Figures 4D,E), respectively.

**TABLE 3:** Montreal Neurological Institute peak coordinates of clusters showing significant psychophysiological interactions for the two task conditions relative to control (*p* < 0.05 FWE at the cluster level).

**Cluster location**	***z*-score**	**Peak coordinates**			***K*-size**
		**X**	**Y**	**Z**	
***Practical help > control condition***					
Medial frontal gyrus	–4.56	–6	15	51	475
Inferior frontal gyrus	–4.51	–54	36	0	358
Middle temporal gyrus	–4.47	–60	–33	–12	122
Middle temporal gyrus	–4.39	63	–42	–9	134
Postcentral gyrus	–4.13	–33	–27	–48	252
Inferior parietal lobule	–3.84	54	–60	27	86
***Effort appreciation > control condition***					
Lingual gyrus	4.42	3	–87	–9	267
Middle frontal gyrus	–4.69	45	36	27	565
Anterior cingulate	–4.04	3	39	24	260

*The negative *Z*-score indicates a reduction of the functional connectivity during the task condition.*

#### Conjunction Analysis

**FIGURE 5:**
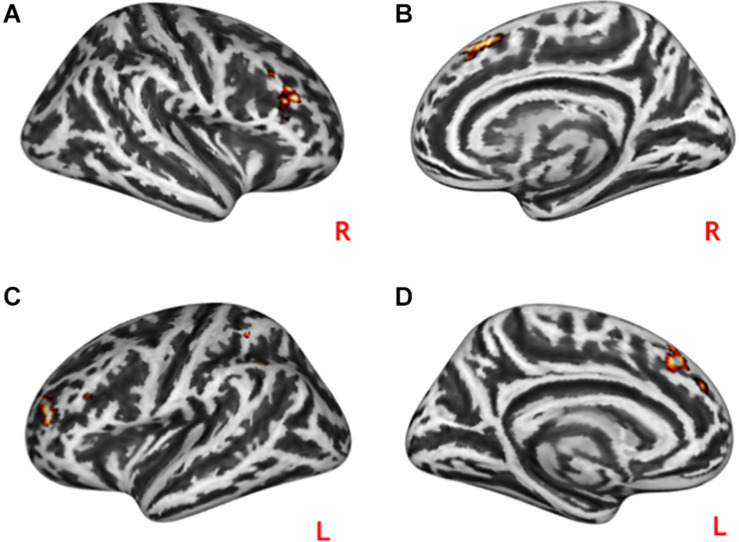
Conjunction analyses between task-modulated connectivity during social interactions. Both practical help and effort appreciation were associated with reduced task-modulated connectivity (PPI) in both the right **(A)**/left **(C)** middle frontal gyrus and right **(B)**/left medial frontal gyrus **(D)**. Statistical probability maps are rendered on an MNI template with a threshold of voxel-wise *p* < 10^–6. MNI, Montreal Neurological Institute. L and R indicate the left and right brain hemisphere, respectively.

Both practical help and effort appreciation were associated with reduced task-modulated connectivity in the right (*t* = 14.33, *p* = 1.0^∗^10^–6^) and left (*t* = 17.20, *p* = 1.0^∗^10^–6^) middle frontal gyrus and right (*t* = 13.56, *p* = 1.0^∗^10^–6^) and left (*t* = 14.84, *p* = 1.0^∗^10^–6^) medial frontal gyrus (see Figure 5).

### Brain Behavior Correlations

Brain activation during practical help was significantly correlated with the C-score for this condition in two clusters within the rMTOC (Supplementary Figure S3): one encompassed the posterior portion of the right middle temporal gyrus and the anterior portion of the right inferior occipital gyrus (xyz = 54, –66, 9; *r* = 0.42; *p* = 0.02), and the second spanned between the right posterior middle temporal gyrus and the angular gyrus (xyz = 45, –60, 12; *r* = 0.43; *p* = 0.02). During effort appreciation, a cluster in the right lingual gyrus showed a significant correlation with the C-score during this condition (xyz = 3, –84, –9; *r* = 0.43; *p* = 0.02). We did not find any correlation between performance and the activation in other clusters that showed an effect of prosocial condition. All the brain behavior correlations did not survive multiple comparison correction using Bonferroni. PPIs did not show any correlation with C-scores for any task condition. We did not find any correlation with any BFQ dimension with the signal of the clusters with significant activations and PPIs.

## Discussion

The present study indicates that receiving unconditional help is associated with the activation of distinct brain regions depending on the type of prosocial aid. During practical help, the medial prefrontal cortex, precuneus as well as bilateral temporo-occipital cortices showed greater activation and reduced task-modulated functional connectivity within a prefrontal-parieto-temporal network. Effort appreciation was associated with the activation of the visual cortex along with increased task-modulated functional connectivity within the visual cortex. Moreover, during effort appreciation, the functional connectivity between the visual and right prefrontal cortex was reduced. Furthermore, the brain activations in right temporo-occipital cortices during practical help, and in the right lingual gyrus during effort appreciation, respectively, were correlated with the propensity to attribute a positive response during social interactions.

Practical help entails direct mentalization of the person who provides help, which includes focusing attention, perspective-taking and estimating the cost-benefit ratio of a behavior (Schilbach et al., 2008; Mars et al., 2012). Practical help was associated with increased activation of the mentalization network, including TPJ, TP, pSTS, mPFC, and PCC activation. Earlier literature has suggested a role of these regions in perspective-taking, in attributing the intent of other people’s actions (Decety and Lamm, 2007) as well as in the understanding of the kinematics of actions (Saxea and Kanwishera, 2003; Frita and Frith, 2006; Kucyi et al., 2012). In practical help, these regions and pSTS, in particular, can contribute to the estimation of the benefit of an action to one person through the updating of the expected value representations that are primarily coded by the mPFC (Hampton et al., 2008) as well as via shifting the focus of attention from the self to the others (Mitchell, 2008). The engagement of the entire ToM network is crucial for grasping the effects of receiving practical help and to reason on how the others’ mental states can be affected by a behavior (Mars et al., 2012). Similarly, increased activation in mPFC and greater functional coupling with rTPJ is consistent with the two cognitive roles that this region plays in practical help: social cognitive processing, including mentalization (Castelli et al., 2000; Rameson et al., 2012; Chavez and Heatherton, 2015; Majdandžić et al., 2016), attribution of the self (Schilbach et al., 2008; Mars et al., 2012), and reward processing (Morelli et al., 2015); cognitive processing for the understanding of the social context and in adapting goal-directed behavior to social outcomes (Powers et al., 2015). Additionally, the engagement of parietotemporal regions during practical help can support high-level cognitive and emotional processing that is needed to understand cognitive information associated in social contexts. These findings are in line with previous imaging literature indicating a recruitment of parieto-temporal cortex in cognitive and emotional processes, including focusing attention, memory, language (McKell Carter and Huettel, 2013; Krautheim et al., 2019). In particular, within lateral temporal cortex we found that pMTG activation was associated with the propensity to experience positive affect in vignettes depicting social interactions. This activation has been reported when expectations of social responses are met (Hampton et al., 2008; Hare et al., 2010) and is consistent with the idea that these regions in practical help support not only intention attribution but also contribute to the affective processing associated with affiliative behavior that may support prosocial exchanges in the long run (Masten et al., 2011). Overall, findings suggest that the posterior regions of the ToM network during practical help may contribute to the perception of “feeling understood” and predict the proneness to have a greater closeness with the actor in a social interaction, which is partly mediated by the mPFC (Morelli et al., 2013).

Effort appreciation was associated with increased activation along with increased functional coupling of the occipital cortex. The lack of activation of prefrontal-cortical-striatal regions during effort appreciation may be due to a high-level control condition that activated similarly to these regions. Our study suggests that the visual cortex is a central node for effort appreciation processing and its over-recruitment may be linked with reward processing as indicated by the association between its engagement and the propensity to experience positive affect in vignettes containing social interactions. The role of visual cortex in reward processing during this condition may ultimately drive social learning. Although earlier studies did not provide evidence of the role of the visual cortex in social reward processing, recent fMRI literature has shown that sensory cortices, including the visual cortex, can significantly contribute to its representation (Brosch et al., 2011; Weil et al., 2015), and can promote social evaluation and reward (Achterberg et al., 2016; Makowski et al., 2016). Notably, the representation of rewarded stimuli can provide traces that are fundamental for a future learning process (Fitzgerald et al., 2013; Schiffer et al., 2014). Increased engagement of the visual cortex could be driven by top-down mechanisms led of attentional modulation by reward processing, including social reward, and learning-dependent signals (Kim and Anderson, 2020). In other words, in the context of social interaction, the acknowledgment of the subject’s efforts by other people may facilitate the performance of the efforts themselves, and therefore the probability of repetition, via the enhancement of learning processes (Kim and Anderson, 2020).

At last, we found reduced connectivity of the rTPJ and lingual gyrus with the prefrontal cortex is reduced during both helping behavior conditions. Prefrontal networks are responsible for executive functions and motor planning. Although relevant for social cognitive processes (Wade et al., 2018), these functions may be deactivated during mentalization to support self-referential processing, taking the perspective of others and social evaluations, which are needed in both task conditions (Andrews-Hanna, 2012).

Overall, we found greater recruitment of occipital regions during effort appreciation relative to practical help. Several factors including arousal levels and reward processes could have contributed to these findings. Emotional scenes with high arousal may increase activation of the visual cortex via amygdalar efferents (Sabatinelli et al., 2011) as well as via attentional modulation (Murray and Wojciulik, 2004) that in its turn may depend on the relevance of the scene (Niu et al., 2012). Although these processes may be influenced individually by personality traits, we did not find such a relationship in our study. Additionally, the social reward processing component of the scenes could also affect brain activation in the visual cortex (see above) but is not associated with mentalization (Alkire et al., 2018). On the other hand, practical help involves greater recruitment of the theory of mind network for its mentalizing component, whereby the arousal and rewarding component associated with these scenes may be minimal. Notably, both effort appreciation and practical help are associated with reduced connectivity with the right dorsolateral prefrontal regions during benefiting of prosocial behavior, which may underlie a shared process of reallocating resources to socially-laden interactions rather than cognitive processing (see above). Future studies addressing the relevance of arousal, emotional, and reward processing are warranted.

From a clinical point of view, the results of this study may also contribute to a better understanding of aberrant prosocial behavior and social interaction in autism and schizophrenia (Sasson et al., 2011; Fernandes et al., 2018). Modulation of these brain regions in the context of disorder-specific psychotherapeutic interventions and improved prosocial behavior in patients with autism and schizophrenia could lead to an increase in social functioning and quality of life (Vass et al., 2018; Holopainen et al., 2019). Finally, the function of the identified regions may be used as potential biomarkers in patients with autism and schizophrenia (and in other psychiatric disorders with disturbances of social interaction) in clinical studies.

We acknowledge some limitations of this study. First, our results rely on real-world interactions that were modeled using block design, which have greater ecological power but the limited ability to identify the brain activity associated with each cognitive process implicated during the social interaction. Second, in our study, to reduce the heterogeneity and increase the power to detect a significant effect, we investigated only a limited type of prosocial interactions. Lastly, the sample size of our study may have not been able to detect all the effects associated with prosocial interactions. Nonetheless, this sample size falls in the average range of participants for similar studies in the moral judgment (Garrigan et al., 2016) and theory of mind (Schurz et al., 2014) field. Future studies investigating other types of prosocial interactions and their subprocesses are warranted.

## Conclusion

Taken together, the current findings highlight the pivotal role of the brain regions supporting ToM as well as the visual cortex during receiving prosocial behavior. Overall, our findings suggest that in social situations, the brain activity in these networks reflects not only the match between the individual’s needs and the other’s actions but may also promote the social interaction directly and through learning via positive affect. From a clinical point of view, progress in this research area can contribute to a better understanding of the physician-patient relationship, where the individual can express needs and show efforts of change that prompt the physician to respond. Furthermore, our results can inform the literature on neurodevelopmental disorders, such as autism and schizophrenia, in which social interactions are crucially impaired (Fernandes et al., 2018).

## Data Availability Statement

The datasets presented in this study can be found in online repositories. The names of the repository/repositories and accession number(s) can be found below: https://neurovault.org/collections/9321/, https://osf.io/2gt8m.

## Ethics Statement

The studies involving human participants were reviewed and approved by the Comitato Etico per Parma. The patients/participants provided their written informed consent to participate in this study.

## Author Contributions

FS, AP, AD, and LG conceived of the experiments. DO and JM performed the experiments and data analyses. FS and DO wrote an initial version of the manuscript. All authors contributed to the interpretation of the results, provided comments, and approved of the final version.

## Conflict of Interest

The authors declare that the research was conducted in the absence of any commercial or financial relationships that could be construed as a potential conflict of interest.

## Footnotes

1 http://rfmri.org/dpabi

2 https://www.fil.ion.ucl.ac.uk/spm/software/spm12/

3 Neurosynth.org
