# Supplementary material for: Neural Responses of Benefiting From the Prosocial Exchange: The Effect of Helping Behavior
Source: Front Psychol. 2021 Mar 4;12:606858. doi: 10.3389/fpsyg.2021.606858 (PMC7969530; doi:10.3389/fpsyg.2021.606858)
Supplement: Supplementary file 1 [file Data_Sheet_1.docx]

**Supplementary Materials**

Neural responses of benefitting from the prosocial exchange: the effect of helping-behavior

Daniele Olivo^1,2^, Andrea Di Ciano^3^, Jessica Mauro^2^, Lucia Giudetti^3^, Alan Pampallona^3^, Katharina M. Kubera^4^, Dusan Hirjak^5^, Robert Christian Wolf^4^, Fabio Sambataro ^1,2,6*^

^1^ Department of Neuroscience (DNS), University of Padova, Padua, Italy

^2^ Department of Medicine (DAME), University of Udine, Udine, Italy

^3^ Fondazione Giancarlo Quarta, Milan, Italy

^4^ Center for Psychosocial Medicine, Department of General Psychiatry, University of Heidelberg, Germany

^5^ Department of Psychiatry and Psychotherapy, Central Institute of Mental Health, Medical Faculty, Mannheim, Heidelberg University, Mannheim, Germany

^6^ Padova Neuroscience Center, University of Padova, Padua, Italy

*** Correspondence:**Fabio Sambataro, MD, PhD.

Department of Neuroscience (DNS), University of Padova, Azienda Ospedaliera di Padova
Via Giustiniani, 2 - 35128 Padova, Italy
email: [fabio.sambataro@unipd.it](mailto:fabio.sambataro@unipd.it)

**S.1. Scenarios and stimuli**

GIF index, number of motion verbs, and the sentence length were compared across helping conditions to measure visual complexity (for pictures), textual complexity, and action content (for sentences), respectively. Briefly, the GIF compression is based on the Lempel-Ziv-Welch “loss-less” data compression algorithm that reduces the size of an image file by identifying and eliminating statistical redundancy. The GIF index, which is computed as the percent of the ratio between the size of the compressed and the original file, shows a strong correlation with subjective visual complexity^1^. We did not find any difference (all p’s>0.1) between helping conditions in visual complexity, (GIF index, mean ± SD: Practical help= 0.58±0.04; Effort appreciation=0.57±0.04), action content (number of motion verbs, mean ± SD: Practical help= 1.90±0.85; Effort appreciation=1.60±1.10), and number words (mean ± SD: Practical help= 8.67±3.74; Effort appreciation=9.2±3.73) of the vignettes.

**S.2. Behavioral performance affective rating distribution**

## Positive responses showed high negative skewness for helping conditions and positive skewness for non-helping conditions (Fig. S2A), conversely, negative responses showed high positive skewness for helping conditions and negative skewness for non-helping conditions (Fig. S2B).

**Figure S2: Histograms of response distribution by affect type and social interaction condition.** Density (y-axis) of the rate of binarized affective responses (x-axis) is displayed for the type of response (Positive, A row; Negative, B row) and social interaction conditions (Practical help, first column; Effort appreciation, second column; Control, third column).

## S.3. Five-regressor general linear model

To confirm that our results were not driven by brain processes other than those associated with prosocial behavior, we also modeled the task, limiting the conditions of interest to the parts in which most of the social cognitive and affective assessments may occur (3^rd^ scene and 3^rd^ vignette). As already described in the methods section, each trial consisted of two parts: a passive presentation of social interactions and an active affect rating task. Data were analyzed using a general linear model with statistical parametrical mapping (SPM12, <https://www.fil.ion.ucl.ac.uk/spm/software/spm12/>). In this case, five regressors were built: one for the introductory part, including the first two blocks of sentences and vignettes (similar for textual and visual complexity in every trial and every scenario), one for each type of social interaction condition (including the the last sentence and last vignette of practical help, effort appreciation and control condition, respectively), and another for the affect rating task were modeled with boxcar functions and convolved with a canonical hemodynamic response function. Six-motion parameters estimated during realignment were included as nuisance covariates. In the first level analyses, whole-brain t-contrast maps of each social interaction condition relative to a non-helping control (practical help> control condition; effort appreciation> control condition) as well as a direct comparison among them (effort appreciation> practical help; practical help> effort appreciation) were computed for each subject. In second-level analyses, individual contrasts were entered in random-effect group analysis^2^ and one-sample t-test spatial maps, depicting the group effect, were obtained. Consistently with the main analyses Family Wise Error-corrected threshold of p<0.05 at the cluster level was applied.

*Main effect of social interaction condition.* During practical help, the inferior region of the temporo-occipital cortex, posterior superior temporal gyrus (pSTG), temporo-parietal junction (TPJ), precuneus, medial postcentral gyrus, and medial prefrontal cortex (mPFC) showed greater activation relative to the control condition (Figure S2.A, B, C, D). Effort appreciation revealed greater activation of the visual cortex relative to the control condition, particularly the lingual gyrus (Figure S2.D, E).

**Figure S2.** **Neural effects of social interactions.** Practical help was associated with increased activation in the inferior region of temporal-occipital cortex, posterior superior temporal gyrus, temporal-parietal junction, precuneus, cingulate cortex, and medial postcentral and prefrontal cortex in both the right (A, D) and left (B, C) hemispheres. Effort appreciation was associated with increased left (E) and right (F) occipital activation. Statistical probability maps are rendered on an MNI template with a threshold of voxel-wise p<0.001 and FWE-corrected p<0.05 at the cluster level. MNI, Montreal Neurological Institute. FWE, family-wise error. L and R indicate the left and right brain hemisphere, respectively.

*Between conditions comparisons*. Practical help showed greater activation in the posterior regions of the temporo-occipital cortex (with a greater extension to the right), dmPFC, precuneus but also in the right temporoparietal junction (TPJ), inferior frontal gyrus (IFG), dorsal pre-motor cortex (dPMC) relative to effort appreciation (Figure S3.A, B, C, D). The visual cortex, particularly the lingual gyrus, showed greater activation during effort appreciation compared to practical help (Figure S3.E, F).

**Figure S3. Neural differences between social interaction conditions.** Practical help showed greater activation in the posterior regions of the temporal-occi pital cortex (with a greater extension to the right), dmPFC, precuneus but also in the right temporoparietal junction (TPJ), inferior frontal gyrus (IFG), dorsal pre-motor cortex (dPMC) relative to effort appreciation (Figure S3.A, B, C, D). The visual cortex, particularly the lingual gyrus, showed greater activation during effort appreciation compared to practical help (Figure S3.E, F). Statistical probability maps are rendered on an MNI template with a threshold of voxel-wise p<0.001 and FWE-corrected p<0.05 at the cluster level. MNI, Montreal Neurological Institute. FWE, family-wise error. TPJ, temporoparietal junction. L and R indicate the left and right brain hemispheres, respectively.

Overall, our results calculated using the whole series of scenes and texts were replicated in a five regressor model where the contrasts of interest were limited to the last scene and text.

## S.3. Brain-behavior correlations

C-score was correlated with activation from significant clusters of activation for the helping conditions as well as for the psychophysiological interactions.

Brain activation during practical help was significantly correlated with the C-score for this condition in two clusters within the rMTOC (Figure. S3): one encompassed the posterior portion of the right middle temporal gyrus and the anterior portion of the right inferior occipital gyrus (xyz= 54, -66, 9, r=0.42, p<0.05), and the second spanned between the right posterior middle temporal gyrus and the angular gyrus (xyz= 45, -60, 12 r=0.43, p<0.05). During effort appreciation, a cluster in the right lingual gyrus showed a significant correlation with the C-score during this condition (xyz= 3 -84 -9, r=0.43, p<0.05). These results did not survive multiple comparison correction using Bonferroni. PPIs did not show any correlation with C-scores for any task condition.


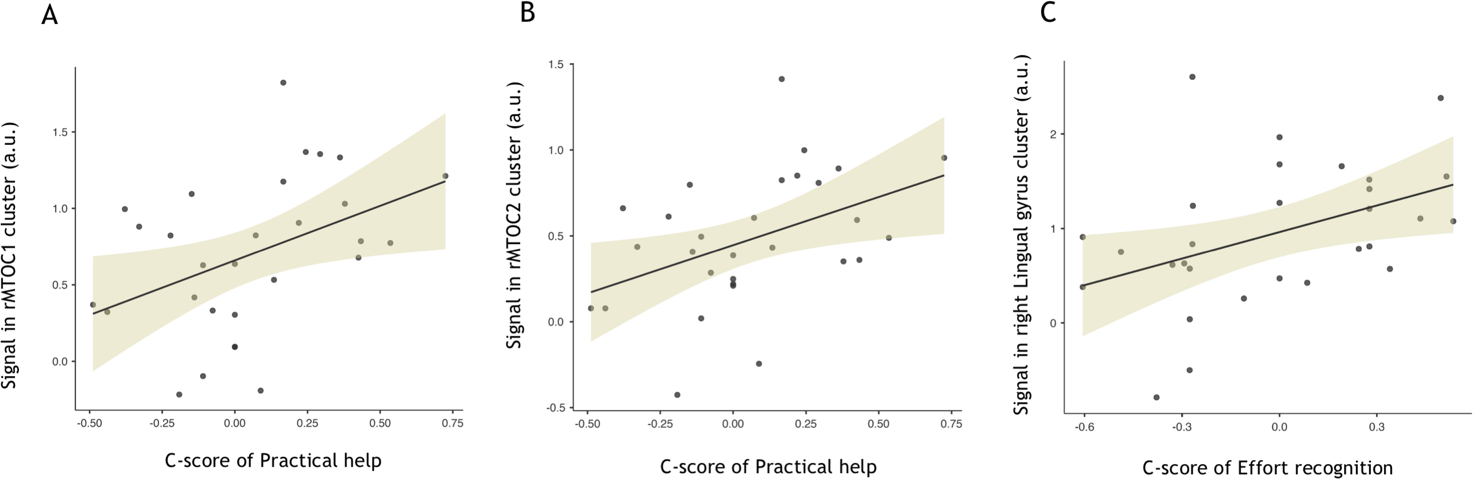
**Figure S3: Scatterplots of brain activation during prosocial conditions and C-scores.** Brain responses during practical help were correlated with C-scores in a cluster encompassing the posterior portion of the right MTG and the anterior portion of the right IOG (rMTOC1; xyz= 54, -66, 9; r=0.42, p<0.05; A) and in another cluster spanning across the right posterior MTG and the AG (rMTOC2; xyz= 45, -60, 12; r=0.43, p<0.05; B); Brain responses during effort appreciation correlated with C-scores in a cluster in the right lingual gyrus (xyz= 3 -84 -9; r=0.43, p<0.05; C). The BOLD signal was extracted, at a group level, using the first eigenvariate from clusters showing a significant effect of the social interaction condition. rMTOC, right middle temporal occipital cortex. MTG, middle temporal gyrus. IOG, inferior occipital gyrus. AG, angular gyrus. BOLD, blood-oxygen-level-dependent.

## S.4. Functional decoding

To infer the mental processes associated with activation and connectivity in specific brain regions from published fMRI studies, a functional decoding approach implemented in Neurosynth database (Neurosynth.org) was used. Briefly, the contrast maps for each prosocial condition and PPI was uploaded to the database and voxel-wise Pearson’s correlations with each of the 400 topic-based meta-analysis maps were carried out (see Yarkoni et al., 2011, for details on Neurosynth methods3). The 10 highest correlated items for each map are reported (Tab S.1, Tbs.2)

**Tab. S.1. Activation functional decoding.**

| **PH>controls** | | **ER>control** |  |  | **PH>ER** | | **ER>PH** | |
| --- | --- | --- | --- | --- | --- | --- | --- | --- |
| **NS term** | **corr.** | **NS term** | **corr.** |  | **NS term** | **corr.** | **NS term** | **corr.** |
| motion | 0.477 | v1 | 0.218 |  | precuneus | 0.335 | precuneus | 0.335 |
| mt | 0.405 | occipital | 0.204 |  | junction | 0.297 | junction | 0.297 |
| v5 | 0.395 | visual | 0.195 |  | motion | 0.271 | motion | 0.271 |
| visual | 0.389 | visual cortex | 0.191 |  | temporoparietal | 0.232 | temporoparietal | 0.232 |
| visual motion | 0.377 | lingual gyrus | 0.181 |  | parietal junction | 0.229 | parietal junction | 0.229 |
| occipital | 0.357 | lingual | 0.163 |  | temporoparietal junction | 0.226 | temporoparietal junction | 0.226 |
| lateral occipital | 0.342 | cerebellar | 0.158 |  | tpj | 0.22 | tpj | 0.22 |
| extrastriate | 0.307 | cerebellum | 0.157 |  | temporo parietal | 0.217 | temporo parietal | 0.217 |
| v1 | 0.289 | fusiform | 0.152 |  | temporo | 0.212 | temporo | 0.212 |
| vision | 0.284 | primary visual | 0.146 |  | theory mind | 0.197 | theory mind | 0.197 |

PH, practical help, ER, Effort recognition, NS, Neurosynth; corr., Pearson correlation with Neurosynth maps

**Tab. S.2. PPI functional decoding.**

| **PPI_PH-** | | **PPI_ER+** | | **PPI_ER-** | | **PPI_Conjunction** | |
| --- | --- | --- | --- | --- | --- | --- | --- |
| **NS term** | **corr.** | **NS term** | **corr.** | **NS term** | **corr.** | **NS term** | **corr.** |
| frontal | 0.296 | v1 | 0.28 | dorsolateral prefrontal | 0.274 | lateral prefrontal | 0.165 |
| tasks | 0.272 | occipital | 0.223 | dorsolateral | 0.268 | medial frontal | 0.161 |
| inferior | 0.271 | visual cortex | 0.201 | prefrontal | 0.227 | anterior prefrontal | 0.15 |
| inferior frontal | 0.271 | visual | 0.188 | dlpfc | 0.205 | monitoring | 0.106 |
| task | 0.27 | early visual | 0.186 | prefrontal cortex | 0.189 | executive | 0.086 |
| parietal | 0.239 | primary visual | 0.167 | cortex dlpfc | 0.163 | dorsolateral | 0.078 |
| working memory | 0.239 | lingual gyrus | 0.15 | lateral prefrontal | 0.151 | prefrontal | 0.075 |
| working | 0.236 | occipital cortex | 0.146 | executive | 0.145 | success | 0.071 |
| language | 0.227 | lingual | 0.14 | cortex acc | 0.144 | prefrontal cortex | 0.066 |
| demands | 0.221 | vi | 0.128 | cognitive | 0.144 | strategic | 0.066 |

PH, practical help, ER, Effort recognition, NS, Neurosynth; corr., Pearson correlation with Neurosynth maps

## S.5. Brain-behavior correlation

We used G*Power 3.1.9.7 to estimate the sample size needed to identify statistically significant differences. Given a moderate effect size of 0.5 (which is consistent with a meta-analysis on scenario paradigms on the theory of mind^4^), the sample size needed to achieve a power of 0.8 with a one-tailed alpha of 0.05, and a statistical model comparing the difference of two means belonging to the same sample is 26.


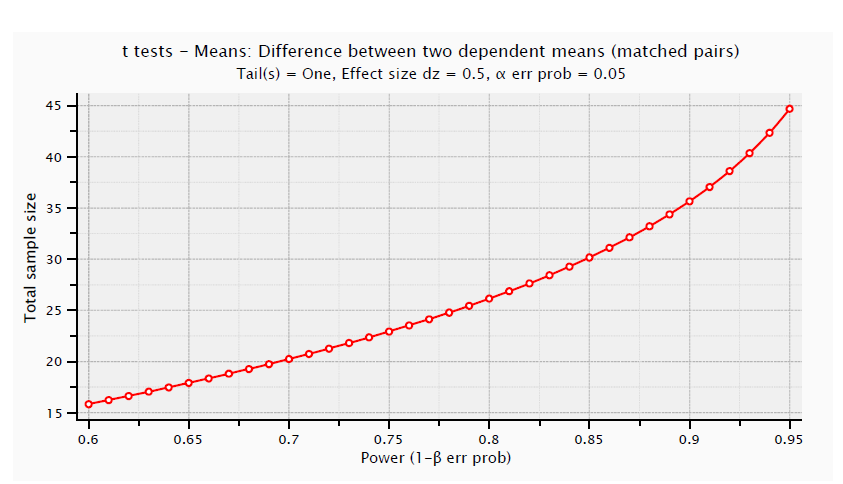


This estimate is consistent with the fMRI literature on the neural responses during moral judgment scenarios meta-analyzed in the work of Garrigan et al.^5^ that reported an average sample size of 26 and 22 for moral evaluations and moral response vignette decisions, respectively, and of Schurtz et al.^4^ on the theory of mind where a sample size of 23 was reported for studies using vignettes.

**S.6. Effect of participant’s-responder’s sex match/mismatch**

To test whether the participant’s sex could influence the affect ratings, we used three-way ANOVAs with participant’s sex, task condition, recipient’s sex as predictors, and positive affect ratings as the dependent variable. *Participant’s sex* * *recipient’s sex* interaction [F(1,111)= 0.0846, P=0.772] and *participant’s sex* * *task condition* * *recipient’s sex* interaction [F(1,111)= 0.2756, P=0.601] were not significant for positive affect ratings. Similarly, *participant’s sex* * *recipient’s sex* interaction [F(1,112)= 0.0935, P=0.760] and *participant’s sex* **task condition* **recipient’s sex* interaction [F(1,112)= 0.0610, P=0.805], were not significant for negative affect ratings. Based on these results, we could exclude the effect of sex match/mismatch in our behavioral task.

**References**

1. Palumbo, L., Makin, A. D. J. & Bertamini, M. Examining visual complexity and its influence on perceived duration Time perception and biases : The role of image complexity. *J. Vis.* **14**, 1–18 (2017).

2. Friston, K. J., Holmes, A. P., Price, C. J., Büchel, C. & Worsley, K. J. Multisubject fMRI studies and conjunction analyses. *Neuroimage* **10**, 385–396 (1999).

3. Yarkoni, T., Poldrack, R. A., Nichols, T. E., Van Essen, D. C. & Wager, T. D. Large-scale automated synthesis of human functional neuroimaging data. *Nat. Methods* **8**, 665–670 (2011).

4. Schurz, M., Radua, J., Aichhorn, M., Richlan, F. & Perner, J. Fractionating theory of mind: A meta-analysis of functional brain imaging studies. *Neurosci. Biobehav. Rev.* **42**, 9–34 (2014).

5. Garrigan, B., Adlam, A. L. R. & Langdon, P. E. The neural correlates of moral decision-making: A systematic review and meta-analysis of moral evaluations and response decision judgments. *Brain Cogn.* **108**, 88–97 (2016).
